# Editorial: Data science and artificial intelligence for (better) science

**DOI:** 10.3389/frma.2023.1177903

**Published:** 2023-04-24

**Authors:** Jean-Claude Burgelman, Kuansan Wang

**Affiliations:** ^1^Department of Social Sciences and Solvay Business School, Open Science, Vrije Universiteit, Brussels, Belgium; ^2^Microsoft Research, Redmond, WA, United States

**Keywords:** data science, artificial intelligence, open data, research life cycle, knowledge production

The impact of data science and AI on science and knowledge production is an important and timely topic. The Frontiers Research Topic entitled “*Data science and artificial intelligence for (better) science*” has collated unique mixes of various contributions from experts, exploring a range of novel approaches to help solving problems facing scientists and advance scientific goals.

Research is also urgent to track the use of open data and to develop approaches to address the opacity of algorithms through open data. Scientific disciplines are called to make data in a way that is findable, accessible, interoperable, and reusable (FAIR), and crosses scientific boundaries.

Meaningful and explainable AI in research can only be fulfilled when as much data as possible is made FAIR (Findable, Accessible, Interoperable, and Reusable). How meaning is communicated in science “as precisely as possible” to machines when we formulate scientific concepts is a key question. Machine readability and interpretability is needed in order to make data and information “Fully AI-Ready” and support data-intensive research (Schultes et al.). The future of science is where there is only “one computer” and FAIR services see all FAIR data and effectively access a global FAIR database.

The single most important challenge is whether Data Science (and AI) can have a key role to improve the credibility and efficiency of research, one of the cornerstones on which science is built. When it comes to research software, caching (Schubotz et al.) can make experiments in research software reproducible. It is also a step forward toward making data related to research software FAIRer by extension.

Questions that science needs to raise with regard to Data Science, for instance, how to interact with data (which includes complex metadata), and how data science can facilitate the scientific cycle (exploration, analysis, interpretation, communication). Predictive models for Web-Enabled scientific discoveries are enabled by the surge of big (social) data. The social data are used to either discover or test scientific hypotheses. The process of scientific discovery is a cyclically sequence of exploration, prediction and validation. Data-driven computational social network science (DD-CSNS) (Emmert-Streib and Dehmer), combining big social data with social networks will enable scientific discoveries.

Finally, the question is how to enable (better) open science. Increasingly relevant today than ever before is the greater reliance on access to data, artificial intelligence (AI) and machine learning (ML). Data access increasingly determines scientific discoveries and advancements. Data reuse is at the forefront of an emerging “third wave of open data” (Verhulst et al., 2020). But despite progress in implementing open data and FAIR principles, science data asymmetries (as in disparities in access to science data) are a growing problem and can undermine scientific progress. Comparative research is needed to document (Verhulst and Young) for instance, investigating the creation of new types of data asymmetries by, e.g., new private-sector investments in data platforms and knowledge repositories, how data portability and interoperability impact the practice of data collaboration, the relationship and interplay between existing asymmetries and technological and societal drivers. Finally, new methods for achieving a social license for data use and reuse toward the public good are needed, capturing multiple stakeholders' acceptance of standard practices and procedures.

## Author contributions

All authors listed have made a substantial, direct, and intellectual contribution to the work and approved it for publication.
